# Effects of Temperature and Nitrogen Application on Carbon and Nitrogen Accumulation and Bacterial Community Composition in Apple Rhizosphere Soil

**DOI:** 10.3389/fpls.2022.859395

**Published:** 2022-04-04

**Authors:** Huanhuan Zhang, Fesobi Olumide Phillip, Linnan Wu, Fengyun Zhao, Songlin Yu, Kun Yu

**Affiliations:** The Key Laboratory of Characteristics of Fruit and Vegetable Cultivation and Utilization of Germplasm Resources of the Xinjiang Production and Construction Corps, Shihezi University, Xinjiang, China

**Keywords:** temperature stress, rhizosphere soil, carbon and nitrogen isotope, bacterial community, *Malus sieversii*

## Abstract

Malus sieversii grows on the slopes of the Tianshan Mountains in Xinjiang where the difference in daily temperature is significant. In recent years, the rhizosphere soil health of *Malus sieversii* has been severely impacted by anthropogenic disturbance and pathogenic infestation. The soil nutrient content and soil microorganism diversity are the main components of soil health. Low temperature has negative effects on soil bacterial community structure by inhibiting the accumulation of carbon and nitrogen. However, the effects of temperature and nitrogen application on soil carbon and nitrogen accumulation and the bacterial community composition in the rhizosphere soil of *Malus sieversii* are unclear. We set two temperature levels, i.e., low temperature (L) and room temperature (R), combined with no nitrogen (N_0_) and nitrogen application (N_1_) to explore the response of plant carbon and nitrogen uptake, rhizosphere soil carbon and nitrogen accumulation and bacterial community composition to temperature and nitrogen fertilization. At the same temperature level, plant ^13^C abundance (P-Atom^13^C), plant ^15^N absolute abundance (P-Con^15^N), soil ^15^N abundance (S-Atom^15^N) and soil urease, protease and glutaminase activities were significantly higher under nitrogen application compared with the no-nitrogen application treatment. The bacterial community diversity and richness indices of the apple rhizosphere soil in the N_1_ treatment were higher than those in the N_0_ treatment. The relative abundances of *Actinobacteria*, *Rhodopseudomonas*, and *Bradyrhizobium* were higher in the LN_1_ treatment than in the LN_0_ treatment. Redundancy analysis (RDA) showed that plant ^13^C absolute abundance (P-Con^13^C) and plant ^15^N absolute abundance (P-Con^15^N) were the main factors affecting the soil bacterial community composition. In summary, Nitrogen application can alleviate the effects of low temperature stress on the soil bacterial community and is of benefit for the uptakes of carbon and nitrogen in *Malus sieversii* plants.

## Introduction

Carbon and nitrogen are important nutrients necessary for plant growth, development, and metabolism and are also important factors limiting soil productivity (Chen et al., 2016; Gu et al., 2019). Carbon and nitrogen nutrition have a direct impact on the formation of photosynthetic products, mineral element uptake, and fruit development (Rodriguez-Lovelle and Gaudillère, 2002; Huang et al., 2015; Sivanandhan et al., 2015). The plant carbon pool will be shifted after nitrogen input (Zhou et al., 2021). Thus, it is necessary to further understand the processes of carbon and nitrogen fixation, allocation and transfer in the plant–soil system (Wang et al., 2019; Wang F. et al., 2020). The capacity of plant photosynthetic carbon fixation to nitrogen input varies across ecosystems, and plant carbon and nitrogen contents and photosynthetic carbon fixation capacity change accordingly (Sheel et al., 2012; Yang et al., 2018). A previous study showed that the δ^13^C values of the upper leaves of *Lolium perenne* L. and *Trifolium repens* L. increased rapidly after 2 days of urea addition, while after 12 days, the δ^13^C values decreased (Ambus et al., 2007). Experiments conducted in Californian chaparral showed a significant increase in aboveground carbon and nitrogen storage rates after 4–5 years of nitrogen application (Vourlitis and Hentz, 2016). Furthermore, a study in a pure *Larix principis-rupprechtii* plantation in northern China showed that nitrogen addition can alter soil enzyme activities and further affect soil carbon turnover through microbial regulation (Wu et al., 2019). Temperature is the limiting factor affecting the growth and respiration of soil microorganisms and enzyme dynamics (Steinweg et al., 2012; Zhong et al., 2021). Different ambient temperatures have different effects on the soil carbon and nitrogen cycles as well as plant development and growth (Dierig et al., 2006; Hatfield and Prueger, 2015; Tulina, 2019; Cruz-Paredes et al., 2021; Hasi et al., 2021). Seasonal low temperature or diurnal variation can significantly affect soil carbon and nitrogen nutrient turnover efficiency (Kurihara et al., 2018). Zhang incubated plants for 16 weeks at four temperatures (10, 15, 20, and 25°C) and discovered that temperature can alter plant metabolism and photosynthesis, as well as the compositions and concentrations of carbon and nitrogen sources, thereby influencing plant δ^13^C and δ^15^N signatures (Zhang P. et al., 2021).

The plant root zone is a soil microzone where plant and microbial communication is highly active (Rzehak et al., 2022). The root system of plants can secrete various microbially beneficial vitamins, enzymes, plant growth regulators, and amino acids (Mommer et al., 2016; Chamkhi et al., 2021), which in turn have an impact on the species, number and distribution of rhizosphere microorganisms (Vives-Peris et al., 2020). The interrelationship of plants, soil, and microorganisms maintains the function of the soil ecosystem (Nihorimbere et al., 2011; Lozano et al., 2014; Purahong et al., 2018; Sun et al., 2018). Soil microorganisms play an important role in the agricultural response to changing ecological environment due to their various nutrient cycles and soil carbon sequestration (Basu et al., 2020; Chen et al., 2021b). The study of the composition of soil microbial communities not only contributes to a more in-depth understanding of the ecological process, but it also has important implications for the conservation of wild resources (Samuel, 2014; Rigg et al., 2016; Shao et al., 2020). Changes in the abundance and diversity of bacteria and in the structural and compositional characteristics of the community can affect soil fertility and the sustainable productivity of fruit forests (Brussaard et al., 2007; Bhat, 2013). Bacteria are an important community of soil microorganism, involved in processes such as nutrient cycling, litter degradation, and soil fertility changes (Cao H. et al., 2021). Nitrogen input has been shown to alter soil nitrogen cycling processes, affecting soil nitrification and denitrification (Li et al., 2010; Yang et al., 2020) and leading to changes in the soil bacterial community structure (Fierer et al., 2021; Xiao et al., 2021). Studies have shown that nitrogen application can significantly increase bacterial abundance (Liu et al., 2020) and alter fungal-to-bacterial ratios (Chinta et al., 2021; Li et al., 2021c), thereby changing the soil microbial community structure and affecting ecosystem biogeochemical cycles (Yu et al., 2021). Nonetheless, numerous studies have found that nitrogen application reduces soil bacterial abundance and diversity (Wang C. et al., 2018; Castellano-Hinojosa et al., 2020; Wang W. et al., 2020). Currently, the effect of nitrogen input on soil bacterial diversity and community composition is still controversial.

*Malus sieversii* (Ledeb.) M. Roem., also known as Tienshan or Xinjiang wide apple, is an important wild fruit tree resource in China (Sitpayeva et al., 2020). It is the original ancestor of the world’s cultivated apples (Harris et al., 2002; Chen et al., 2007) and is listed as a second-class priority plant in China and a national biodiversity priority species (Fu and Chin, 1992). The genetic resources of *M*. *sieversii* are rich and diverse and are of great value in the conservation and utilization of germplasm resources (Wiedow et al., 2004; Wang N. et al., 2018). There are numerous links between plant and soil microbial diversity, and plant species and microbial diversity both play important roles in maintaining ecosystem stability and health (Zak et al., 2003; Gabriele et al., 2017; Rawat et al., 2020). Numerous scholars have conducted systematic studies on the response of diversity to environmental changes and investigated the feedback mechanisms of plant species and microbial diversity (Bouasria et al., 2012; Oliveira et al., 2012; Jia et al., 2021; Li et al., 2021a). Currently, it is extremely difficult to replace new populations of *M*. *sieversii* with live seedlings under natural conditions (Liu and Dong, 2018). Therefore, it is particularly important to study the response of soil carbon and nitrogen allocation to temperature and nitrogen fertilization and their microbial mechanisms. In this study, ^13^C and ^15^N isotope dual-labeling technology and Illumina NovaSeq high-throughput sequencing technology were used to explore differences in the rhizosphere soil carbon and nitrogen distribution and bacterial community diversity. This study provides fundamental information for the dynamic balance of rhizosphere soil ecology in *M*. *sieversii*, thereby providing new insights into plant–soil–microbe interactions that can be harnessed for *M*. *sieversii* seedlings breeding and germplasm conservation.

## Materials and Methods

### Experimental Design

Our experiment was conducted in the Key Laboratory of Special Fruits and Vegetables Cultivation Physiology and Germplasm Resources Utilization of Xinjiang Production and Construction Corps of Shihezi University, Xinjiang Uygur Autonomous Region, China. The *M*. *sieversii* seeds were treated with low-temperature lamination at 4°C for 90 days under dark conditions. On November 11, 2019, 200 seeds with consistent germination growth were selected and sown in 50-cell seedling trays containing a mixed substrate with peat–vermiculite–apple orchard soil (volume ratio 3:1:0.2, and the peat–vermiculite mixture was autoclaved). One plant was grown per cell, and each cell of the tray measured 4 cm in length, 4 cm in width and 10 cm in height. *M*. *sieversii* seeds were incubated in an artificial climate chamber (*RXZ-300B*, *Ningbo Jiangnan Instrument Co*., Ningbo, China). The culture conditions were as follows: temperature 25°C, relative humidity 70–80%, darkness during the germination period, light intensity 134 μmol m^–2^ s^–1^ during the seedling emergence period, and a 12-h:12-h light-dark cycle. Individual, healthy and uniform seedlings (when they had 7–8 true leaves) were selected for isotope labeling and low temperature treatment. The seedlings were watered once every 3 days during planting period as needed (Nagakura et al., 2004; Fernandez-Going et al., 2013).

**FIGURE 1:**
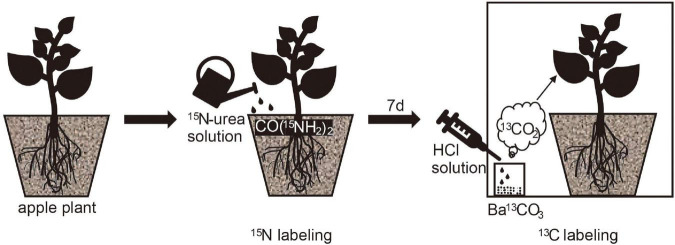
The ^13^C and ^15^N double isotope labeling experiment.

A solution containing 320 mg CO(^15^NH4)_2_ (abundance of 10.16%) was dissolved in water and was applied to burrowing trays on January 3, 2020. Nitrogen labeling was performed 7 days after ^13^C pulse labeling, and labeling was performed in a transparent agricultural film labeling chamber (Figure 1). The seal of the marker chamber was checked before marking. A syringe was used to inject 1 mL of HCl solution at a concentration of 1 mol/L into a test tube containing 0.6 g of Ba^13^CO_3_ (abundance of 98%). Two nitrogen fertilizer treatments were set up, i.e., the N_1_ treatment (urea applied at a fertilizer to substrate ratio of 0.43 g kg^−1^; N_1_) and N_0_ treatment (0 g kg^−1^; N_0_). The temperature was set at two levels, i.e., the L treatment (5°) and R treatment (25°). The experiment was designed based on a completely randomized design with four treatment groups (LN_0_, LN_1_, RN_0_, and RN_1_) and three replications per treatment.

### Sample Collection

Samples were collected on the 7th day of the low temperature and nitrogen application treatments. Three *M*. *sieversii* seedlings were randomly selected from each of the four treatments, the aboveground parts were cut off, and the plants were destructively removed. Large clods of soil around the root system were removed, and the soil was gently shaken off the root surface of the plants. A portion of the collected fresh samples was directly packed into sterile plastic bags and stored at −80°C in the refrigerator for soil DNA extraction. The other part was mixed well and dried naturally, ground through a 0.25 mm sieve and placed in plastic bags for the determination of soil enzyme activity and soil ^13^C and ^15^N abundance. Three additional *M*. *sieversii* seedlings with essentially uniform growth were randomly selected for destructive sampling in each treatment. The samples were rinsed in the order of water, detergent, water, 1% hydrochloric acid and then three times with deionized water, after which they were dried at 105°C for 30 min, followed by drying at 80°C to a constant weight (Zhang R. et al., 2021). The dried samples were ground through a 0.25 mm sieve and stored in plastic bags for determination of the ^13^C and ^15^N abundance of the plants (Yan et al., 2020).

### ^13^C and ^15^N Abundance

The ^13^C and ^15^N abundances were measured using a *DELTA V* advantage isotope ratio mass spectrometer and were analyzed by the China Academy of Forestry Sciences Stable Isotope Laboratory. The formulas were as follows: P-Atom^13^C (or S-Atom^13^C) = (δ^13^C + 1,000) × R_*PDB*_/[(δ^13^C + 1,000) × R_*PDB*_ + 1,000] × 100, P-Con^13^C (or S-Con^13^C) = P-Atom^13^C (or S-Atom^13^C) × 0.01 × C% × 0.01 × 1,000, P-Atom^15^N (or S-Atom^15^N) = (δ^15^N + 1,000) × R_*PDB*_/[(δ^15^N + 1,000) × R_*PDB*_ + 1,000] × 100, P-Con^15^N (or S-Con^15^N) = P-Atom^15^N (or S-Atom^15^N) × 0.01 × N% × 0.01 × 1,000, and δ^13^C (or δ^15^N) = (Rs/R_*PDB*_ − 1) × 1,000, where δ^13^C is the amount of ^13^C assimilate that was fixed (‰); δ^15^N is the amount of ^15^N assimilate that was fixed (‰); R_*S*_ is the ratio of ^13^C to ^12^C (or the ratio of ^15^N to ^14^N); R_*PDB*_ is the standard ratio of carbon isotopes, i.e., 0.0112372 (or the standard ratio of nitrogen isotopes, i.e., 0.0036765); P-Atom^13^C and P-Atom^15^N are plant ^13^C abundance and plant ^15^N abundance (%), respectively, which refer to ^13^C and ^15^N as percentages of total carbon and nitrogen of the plant samples, respectively; S-Atom^13^C and S-Atom^15^N are soil ^13^C abundance and soil ^15^N abundance (%), respectively, which refer to ^13^C and ^15^N as percentages of total carbon and nitrogen of the soil samples; P-Con^13^C and P-Con^15^N are plant ^13^C absolute abundance and plant ^15^N absolute abundance (mg g^−1^), respectively, which refer to the amount (mg) of ^13^C and ^15^N contained in one gram of the plant sample; S-Con^13^C and S-Con^15^N are soil ^13^C absolute abundance and soil ^15^N absolute abundance (mg g^−1^), respectively, which refer to the amount (mg) of ^13^C and ^15^N contained in one gram of the soil sample; and C% and N% are the amount (g) of total carbon and nitrogen contained in 100 g of sample.

### Determination of Soil Enzymatic Activities

Determination of rhizosphere soil urease (EC3.5.1.5) and catalase (EC1.11.1.6) activities according to the method described by Guan (1986). Urease activity (EC3.5.1.5) was measured by the colorimetric analysis of sodium phenate-sodium hypochlorite, and the activity was expressed as micrograms of NH^3^-N in 1 g of soil after 24 h (μg g^–1^ d^–1^). Catalase activity (EC1.11.1.6) was evaluated using the potassium permanganate titration volume method, and the activity was expressed as milliliters of 0.1 mol L^−1^ potassium permanganate in 1 g of soil after 20 min (ml g^–1^ 20 min^–1^). Protease activity (EC 3.4.2.21-24) was determined according to the method developed by Ladd and Butler (1972), and the activity was expressed as micrograms of glycine in 1 g of soil after 24 h (μg g^–1^ d^–1^). Glutaminase activity (EC3.5.1.2) was assayed using a glutaminase kit (Beijing Solarbio Science & Technology Co., Ltd., Beijing, China) with the specification of 50 tubes/24 samples. The method was visible spectrophotometry, and 1 g of soil-catalyzed glutamine production of 1 μmol L^−1^ ammonia per day at 37°C was defined as one enzyme activity unit (U g^−1^) (Sakai et al., 2022).

### Soil DNA Extraction, PCR Amplification, and Illumina Sequencing

The genomic DNA of the samples was extracted using the SDS method (Nanasato et al., 2018). The purity and concentration of DNA were subsequently examined using agarose gel electrophoresis. An appropriate amount of sample DNA was placed in a centrifuge tube, and the sample was diluted to 1 ng μL−1 using sterile water. To ensure amplification efficiency and accuracy, PCR amplification of the V4 region gene fragment was performed using primers 515F (5′-GTGCCAGCMGCCGCGGTAA-3′) and 806R (5′-GGACTACHVGGGTWTCTAAT-3′) and high-fidelity polymerase (Carini et al., 2016). The PCR mixture (30 μl) contained 15 μL of Phusion Master Mix, 3 μL of each primer and 10 μL of DNA template (5–10 ng). The amplification program consisted of predenaturation at 98°C for 1 min, 30 cycles (denaturation at 98°C for 10 s, annealing at 50°C for 30 s, extension at 72°C for 30 s), and a final extension step at 72°C for 5 min. The PCR products were extracted from 2% agarose gel, and the target bands were recovered using a gel recovery kit (QIAGEN China Co., Ltd. Guangzhou, China) (Abdel-Ghany et al., 2016). The products were then assayed for quantification and mixing, and library construction was performed after mixing and purification. The qualified libraries were sequenced using an Illumina NovaSeq6000 (Illumina, San Diego, CA, United States) (Modi et al., 2021).

### Bioinformatics Analysis and Data Processing

The data of each sample were split from the downstream data based on barcode sequences and PCR amplification primer sequences. The sequences of barcodes and primers were intercepted and then spliced and filtered using FLASH (Magoc and Salzberg, 2011) and QIIME (Caporaso et al., 2010). The chimeric sequences were removed from these sequences to obtain the final valid data. OTUs were obtained by clustering the sequences with 97% similarity among the valid sequences of all samples using Uparse software (Haas et al., 2011). The SSUrRNA database (Wang et al., 2007) of SILVA132 (Edgar, 2013) was subsequently consulted for species annotation of OTU sequences, and diversity index, species classification and abundance analysis were carried out. In addition, redundancy analysis (RDA) was used to identify key environmental factors that significantly influenced changes in bacterial communities between treatment groups.

Alpha diversity analysis (including Shannon, Simpson, Chao1, and Ace indices) was performed using QIIME (Version 1.9.1). Significant differences between treatments were evaluated by one-way analysis of variance (ANOVA) followed by Tukey’s multiple comparison test using SPASS 20.0 (SPSS Inc., Chicago, IL, United States). Origin 2021 (Origin Software, Inc. Guangzhou, China) was used for plotting. Redundancy analysis (RDA) was used to examine the relationship between the ^13^C and ^15^N abundance of plants and rhizosphere soil and the rhizosphere soil bacterial community compositions with the CANOCO 5.0 software (Microcomputer Power, Ithaca, NY, United States).

## Results

### ^13^C and ^15^N Abundance

**FIGURE 2:**
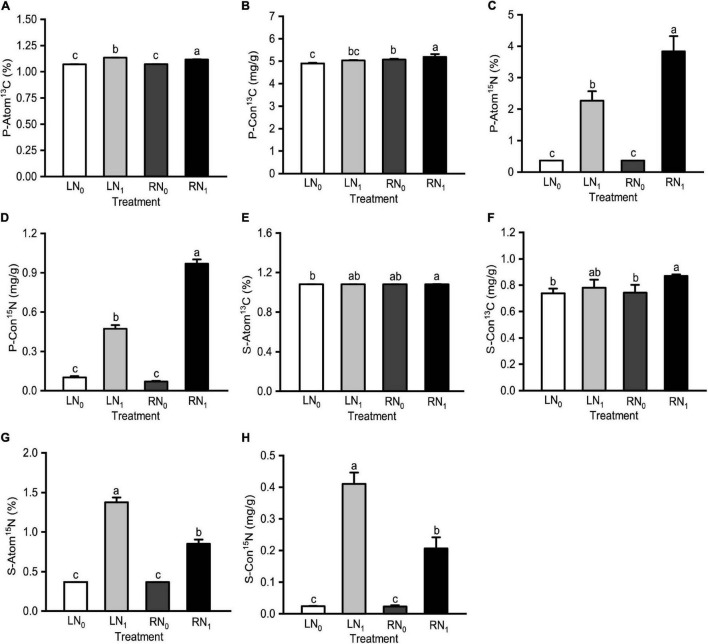
Comparisons of **(A)** plant ^13^C abundance (P-Atom ^13^C), **(B)** plant ^13^C absolute abundance (P-Con^13^C), **(C)** plant ^15^N abundance (P-Atom^15^N), **(D)** plant ^15^N absolute abundance (P-Con^15^N), **(E)** soil ^13^C abundance (S-Atom ^13^C), **(F)** soil ^13^C abundance (S-Con^13^C), **(G)** soil ^15^N absolute abundance (S-Atom^15^N), and **(H)** soil ^15^N absolute abundance (S-Con^15^N) among different treatments. L and R were low and room temperature treatments, N_0_ and N_1_ were non-nitrogen and nitrogen treatments. Values were shown as means ± standard deviations (SD, *n* = 3). Different lowercase letters were indicated statistically significant differences between the four treatments at 0.05 level.

Temperature and nitrogen application treatments significantly affected P-Atom^13^C, P-Con^13^C, P-Atom^15^N, P-Con^15^N, S-Atom^13^C, S-Con^13^C, S-Atom^15^N, and S-Con^15^N (Supplementary Table 1). P-Atom^13^C and P-Atom^15^N were significantly and positively correlated with S-Atom^13^C, S-Con^13^C, S-Atom^15^N and S-Con^15^N (*P* < 0.05) (Supplementary Table 5). P-Atom^13^C, P-Con^13^C, and S-Con^13^C of the RN_1_ treatment were significantly higher than those of the RN_0_ treatment (*P* < 0.05) (Figures 2A,B,F). There was no significant difference between P-Con^13^C, S-Atom^13^C, and S-Con^13^C of the LN_0_ treatment and LN_1_ treatment (*P* > 0.05) (Figures 2B,E,F). P-Atom^15^N and P-Con^15^N of the RN_1_ treatment were significantly higher than those of the LN_1_ treatment by 69.05% and 105.06%, respectively (Figures 2C,D). There was no significant difference between P-Atom^15^N, P-Con^15^N, S-Atom^15^N, and S-Con^15^N in the LN_0_ treatment and RN_0_ treatment (*P* > 0.05) (Figures 2C,D,G,H). S-Atom^15^N and S-Con^15^N of the RN_1_ treatment were significantly lower than those of LN_1_ by 38.25 and 49.63%, respectively (*P* < 0.05) (Figures 2G,H). Both S-Atom^15^N and S-Con^15^N had the following treatment rankings: LN_1_ > RN_1_ > LN_0_, RN_0_.

### Soils Enzymatic Activity

**FIGURE 3:**
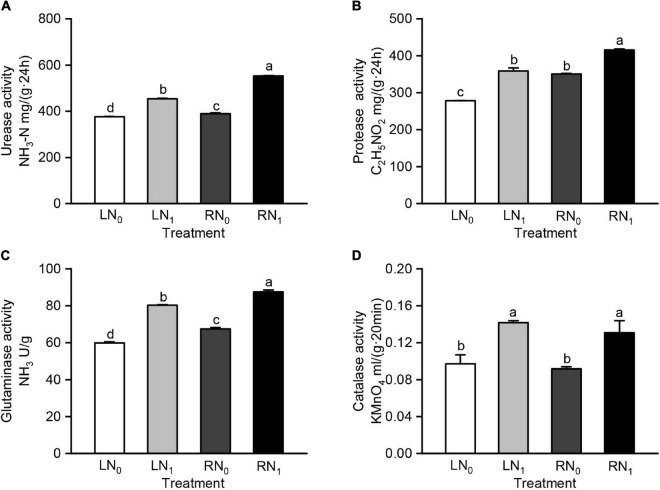
Comparisons of **(A)** soil urease activity, **(B)** soil protease activity, **(C)** soil glutaminase activity, and **(D)** soil catalase activity among different treatments. L and R were low and room temperature treatments, N_0_ and N_1_ were non-nitrogen and nitrogen treatments. Values were shown as means ± standard deviations (SD, *n* = 3). Different lowercase letters were indicated statistically significant differences between the four treatments at 0.05 level.

There were significant differences in the rhizosphere soil urease, protease, and glutaminase catalase activities between the different temperature and nitrogen application treatments (Supplementary Table 1). At the same temperature level, the urease activity was significantly higher in the nitrogen application treatment group (LN_1_, RN_1_) than in the no-nitrogen treatment group (LN_0_, RN_0_) (*P* < 0.05); at the same nitrogen level, the urease activity was significantly higher in the room temperature treatment group (RN_0_, RN_1_) than in the low temperature treatment group (LN_0_, LN_1_) (*P* < 0.05) (Figure 3A). The urease activity of the RN_0_ and RN_1_ treatments was significantly higher than that of the LN_0_ and LN_1_ treatments by 3.49% and 21.95%, respectively (*P* < 0.05). The trends of the protease and glutaminase activities in each treatment were consistent with that of the urease activity (Figures 3B,C). Protease and glutaminase activities were significantly increased by 28.84% and 34.18% in the LN_1_ treatment compared to the LN_0_ treatment (*P* < 0.05). The catalase activity of the LN_1_ and RN_1_ treatments was significantly higher than that of the LN_0_ and RN_0_ treatments by 46.05 and 42.55%, respectively (*P* < 0.05) (Figure 3D).

### Bacterial Community Alpha Diversity

After sequencing quality filtering of the base-called sequences, a total of 738,893 high-quality sequences were obtained for the bacteria. A total of 3,148 operational taxonomic units (OTUs) at 97% similarity were obtained from the rhizosphere soil (Table 1). Temperature and nitrogen application treatments had significant effects on the Shannon, Simpson, Chao1, and Ace indices of the rhizosphere soil bacterial community of *M*. *sieversii* (Supplementary Table 1). Bacterial community diversity indices (Shannon and Simpson indices) showed that the Shannon index of the N_1_ treatment was significantly higher than that of N_0_ treatment (*P* < 0.05), and the Simpson index of the LN_0_ treatment was significantly higher than that of the other three treatments (*P* < 0.05). The Shannon index of the RN_1_ treatment was significantly higher than that of the other treatments (*P* < 0.05), and the Simpson index of the RN_1_ treatment was the lowest (0.976), indicating the highest soil bacterial community diversity in the RN_1_ treatment. Bacterial community richness indices (Chao1 and Ace indices) showed that the Chao1 index of the LN_1_ and RN_1_ treatments was significantly higher than that of LN_0_ and RN_0_ treatments (*P* < 0.05), and the Ace index of the RN_1_ treatment was significantly higher than that of the LN_0_, LN_1_, and RN_0_ treatments by 21.85, 10.64, and 17.70%, respectively (*P* < 0.05).

**TABLE 1:** Effect of temperature and nitrogen application on alpha diversity index in rhizosphere soil bacterial communities of *M*. *sieversii*.

Treatment	Sequences	OTUs	Diversity and richness indexes			
			Shannon	Simpson	Chao1	ACE
LN_0_	58,804 ± 549c	1,510 ± 23c	7.246 ± 0.152d	0.989 ± 0.001a	2,040.597 ± 51.456b	1,648.302 ± 39.603c
LN_1_	61,699 ± 1,097b	1,679 ± 20b	7.546 ± 0.030b	0.977 ± 0.001c	2,294.130 ± 45.581a	1,815.171 ± 19.432b
RN_0_	60,315 ± 120bc	1,617 ± 21bc	7.416 ± 0.031c	0.983 ± 0.002b	2,139.913 ± 76.140b	1,706.402 ± 36.419c
RN_1_	65,480 ± 1218a	1,857 ± 120a	7.667 ± 0.020a	0.976 ± 0.001c	2,418.799 ± 50.862a	2,008.384 ± 60.436a

*Values were shown as means ± standard deviations (SD, n = 3). Different lowercase letters in the same column were indicated statistically significant differences between the four treatments at 0.05 level.*

### Composition of the Bacterial Communities

**FIGURE 4:**
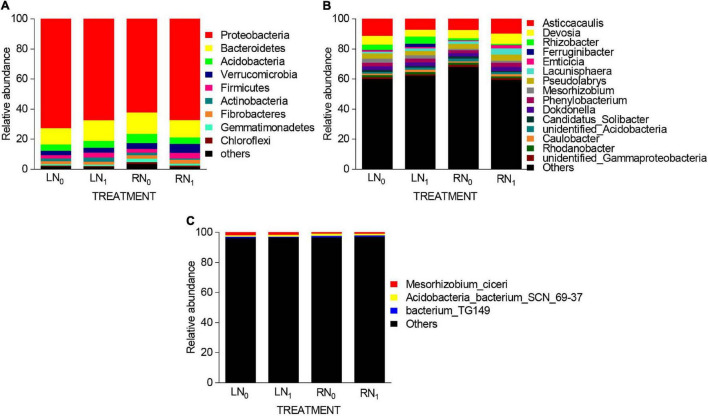
Relative abundance of primary **(A)** bacterial phyla (relative abundance ≥ 0.5%), **(B)** bacterial genera (relative abundance ≥ 0.5%), and **(C)** bacterial species (relative abundance ≥ 0.5%) present in the rhizosphere soil bacterial communities of the different treatments.

At the phylum level, a total of 33 bacterial phyla were obtained, and 9 dominant phyla were obtained (Figure 4A). Among them, the dominant phyla (relative abundance > 5%) were Proteobacteria, Bacteroidetes, Acidobacteria, and Verrucomicrobia. The relative abundances of Proteobacteria, Bacteroidetes, Acidobacteria, and Verrucomicrobia were 62.05–72.59%, 10.89–14.23%, 4.13–6.11%, and 2.92–5.95%, respectively, accounting for 86.37–90.53% of all phyla. The average relative abundance of the other five phyla only accounted for 9.47–13.63% of the total bacterial community. Further analysis of bacterial phyla with relative abundances greater than 1% showed that temperature and nitrogen application treatments at the bacterial phylum level had significant effects on Proteobacteria, Acidobacteria, Verrucomicrobia, and Actinobacteria in the rhizosphere soil (Supplementary Tables 1, 2). The relative abundance of Acidobacteria was significantly higher in the RN_0_ treatment than in the LN_0_ treatment (*P* < 0.05). The relative abundance of Verrucomicrobia in the RN_1_ treatment was 103.45, 84.38, and 47.50% higher than that in the LN_0_, LN_1_, and RN_0_ treatments, respectively. The relative abundance of Actinobacteria in the LN_1_ treatment was significantly higher than that in the RN_0_ and RN_1_ treatments by 58.82% and 170.00%, respectively (*P* < 0.05). At the genus level, a total of 404 bacterial genera were obtained. Fifteen dominant genera with relative abundances greater than 0.5% were obtained in each sample (Figure 4B). The two most abundant bacterial genera in the LN_0_ treatment were *Asticcacaulis* (11.14%) and *Devosia* (5.83%). The relative abundances of *Asticcacaulis* (7.07%) and *Rhizobacter* (4.68%) were highest in the LN_1_ treatment. Moreover, the relative abundance of *Asticcacaulis* was highest in both the RN_0_ and RN_1_ treatments, i.e., 7.28 and 9.55%, respectively. At the species level, a total of 237 bacterial species were obtained. The dominant species (relative abundance > 5%) were *Mesorhizobium_ciceri*, *Acidobacteria_bacterium_SCN_69-37*, and *bacterium_TG149*. Other bacterial species had the largest proportion, with an average relative abundance of 96.76% (Figure 4C).

### Carbon- and Nitrogen-Fixing Bacterial Genera

The relative abundances of *Rhodopseudomonas*, *Methylibium*, *Pseudomonas*, and *Bradyrhizobium* differed significantly between the different temperature and nitrogen fertilization treatments (Supplementary Table 1). The relative abundances of *Rhodopseudomonas* and *Methylibium* were significantly higher in the nitrogen application treatment groups (LN_1_, RN_1_) than in the non-nitrogen application treatment groups (LN_0_, RN_0_) at the same temperature level (Table 2). The relative abundances of *Rhodopseudomonas* and *Methylibium* in the LN_1_ treatment were significantly higher than those in the LN_0_ treatment by 75.00 and 129.03%, respectively (*P* < 0.05). The relative abundances of *Rhodopseudomonas* and *Methylibium* in the RN_1_ treatment were significantly higher than those in the RN_0_ treatment by 120.00% and 178.72%, respectively (*P* < 0.05). The relative abundance of *Pseudomonas* and *Bradyrhizobium* was higher in the nitrogen treatment group (LN_1_, RN_1_) than in the non-nitrogen treatment group (LN_0_, RN_0_) at the same temperature level. The relative abundance of *Bradyrhizobium* was significantly higher in the LN_1_ and RN_1_ treatments than in the LN_0_ and RN_0_ treatments by 63.27% and 81.36%, respectively (*P* < 0.05).

**TABLE 2:** Effect of temperature and nitrogen application on significantly different carbon- and nitrogen-fixing bacterial genera in the rhizosphere soil.

Treatment	*Rhodopseudomonas*	*Methylibium*	*Pseudomonas*	*Bradyrhizobium*
LN_0_	0.0008 ± 0.00013c	0.0031 ± 0.00046c	0.0013 ± 0.00012b	0.0049 ± 0.00026c
LN_1_	0.0014 ± 0.00026b	0.0071 ± 0.00213b	0.0049 ± 0.00085b	0.0080 ± 0.00091b
RN_0_	0.0010 ± 0.00003bc	0.0047 ± 0.00044bc	0.0035 ± 0.00043b	0.0059 ± 0.00082c
RN_1_	0.0022 ± 0.00046a	0.0131 ± 0.00338a	0.0124 ± 0.00585a	0.0107 ± 0.00156a

*Values were shown as means ± standard deviations (SD, n = 3). Different lowercase letters in the same column were indicated statistically significant differences between the four treatments at 0.05 level.*

### Bacterial Phyla and Bacterial Genera and Correlation With Environmental Parameters

**FIGURE 5:**
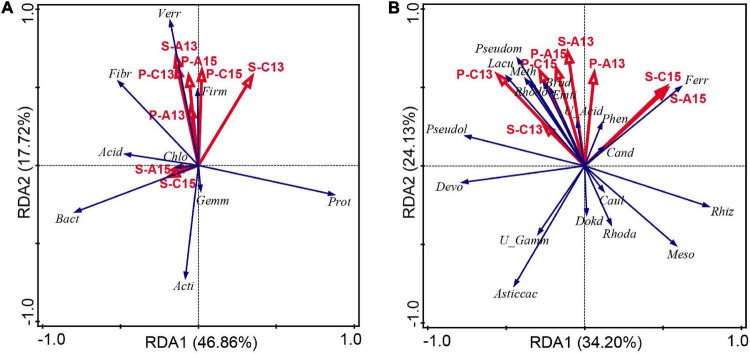
Redundancy analysis of **(A)** dominant bacterial phyla (relative abundance ≥ 0.5%) and **(B)** dominant bacterial genera (relative abundance ≥ 0.5%) and significantly different bacterial genera related to metabolism of carbon and nitrogen across all of the soil samples. Phyla and genera are indicated by blue vectors and environmental variables are represented by red vectors. The positions and lengths of the arrows indicate the directions and strengths, respectively, of the effects of variables on bacterial communities. Abbreviations in panel **(A)**, Acid, Acidobacteria; Acti, Actinobacteria; Bact, Bacteroidetes; Chlo, Chloroflexi; Fib, Fibrobacteres; Firm, Firmicutes; Gemm, Gemmatimonadetes; P-A13, P-Atom^13^C; P-A15, P-Atom^15^N; P-C13, P-Con^13^C; P-C15, P-Con^15^N; Prot, Proteobacteria; S-A13, S-Atom^13^C; S-A15, S-Atom^15^N; S-C13, S-Con^13^C; S-C15, S-Con^15^N; Verr, Verrucomicrobia. Abbreviations in panel **(B)**, Asti, *Asticcacaulis*; Brad, *Bradyrhizobium*; Cand, *Candidatus_Solibacter*; Caul, *Caulobacter*; Devo, *Devosia*; Dokd, *Dokdonella*; Emti, *Emticicia*; Ferr, *Ferruginibacter*; Lacu, *Lacunisphaera*; Meso, *Mesorhizobium*; Meth, *Methylibium*; P-A13, P-Atom^13^C; P-A15, P-Atom^15^N; P-C13, P-Con^13^C; P-C15, P-Con^15^N; Pseudol, *Pseudolabrys*; Pesudom, *Pseudomonas*; Phen, *Phenylobacterium*; Rhiz, *Rhizobacter*; Rhoda, *Rhodanobacter*; Rhodo, *Rhodopseudomonas*; S-A13, S-Atom^13^C; S-A15, S-Atom^15^N; S-C13, S-Con^13^C; S-C15, S-Con^15^N; U_Acid, *unidentified_Acidobacteria*; U_Gamm, *unidentified_Gammaproteobacteria*.

The relationship between plant ^13^C and ^15^N abundance, soil ^13^C and ^15^N abundance and bacterial phyla (relative abundance > 0.5%) in rhizosphere soil was analyzed by RDA. Considering the ^13^C and ^15^N abundance of *M*. *sieversii* plants and rhizosphere soil as environmental variables, axes 1 and 2 explained 46.86% and 17.72%, respectively, of the total variation (Figure 5A). P-Atom^15^N, P-Con^15^N, and S-Atom^13^C were negatively correlated with Proteobacteria, Bacteroidetes, Actinobacteria, Gemmatimonadetes, and Chloroflexi (*P* ≥ 0.05) (Figure 5A and Supplementary Table 3). P-Atom^15^N and P-Con^15^N were significantly and positively correlated with Verrucomicrobia and Firmicutes (*P* < 0.05). The RDA showed that the bacterial communities were differentially influenced by ^13^C and ^15^N abundance. The contribution of P-Con^15^N was 28.00%, which was the environmental factor with the largest contribution.

The relationship between plant ^13^C and ^15^N abundance, soil ^13^C and ^15^N abundance and bacterial genera in rhizosphere soil was analyzed by RDA. Considering the ^13^C and ^15^N abundance of *M*. *sieversii* plants and rhizosphere soil as environmental variables, axes 1 and 2 explained 34.20% and 24.13%, respectively, of the total variation (Figure 5B). P-Atom^13^C, P-Con^13^C, P-Atom^15^N, and P-Con^15^N showed highly significant positive correlations with the carbon and nitrogen metabolism-related bacterial genera *Rhodopseudomonas*, *Methylibium*, and *Bradyrhizobium* (*P* < 0.01) (Figure 5B and Supplementary Table 4). S-Atom^13^C and S-Con^13^C were significantly positively correlated with *Rhodopseudomonas*, *Methylibium*, *Pseudomonas*, and *Bradyrhizobium* (*P* < 0.05). S-Atom^13^C, S-Con^13^C, S-Atom^15^N, and S-Con^15^N were significantly positively correlated with *Caulobacter* (*P* < 0.05). RDA also showed that P-Con^13^C (24.60%) and S-Atom^15^N (24.40%) were the two factors with the highest contribution.

## Discussion

### Rhizosphere Soil Carbon and Nitrogen Portioning

Carbon and nitrogen metabolism are the two most important metabolic processes in plants, and they are very closely related (Nunes-Nesi et al., 2010; Zhang et al., 2018; Zhong et al., 2021). Carbon metabolism provides carbon and energy for nitrogen metabolism, which in turn provides enzymes and photosynthetic pigments for carbon metabolism, both of which together regulate the material and energy metabolic processes in plants (Zhang et al., 2014; Ding et al., 2017). The photosynthetic carbon sequestration capacity of plants in different ecosystems responds in different ways to nitrogen inputs (Zhu et al., 2021). In this study, ^13^C and ^15^N dual-labeled isotope tracing techniques revealed that the ^13^C abundance and ^13^C absolute abundance of *M*. *sieversii* plants with high ^15^N abundance and ^15^N absolute abundance were also at higher levels in the treatments, indicating that appropriate nitrogen levels can promote the allocation and functioning of carbon assimilates. Zhao et al. (2021) showed that increased nitrogen fertilizer application increased photosynthetic carbon accumulation in wheat by 11–20% during 62 consecutive days of ^13^CO_2_ labeling. In this study, the ^13^C abundance and ^13^C absolute abundance of plants and soil showed roughly the same distribution pattern at different temperatures and nitrogen levels. The ^13^C abundance and ^13^C absolute abundance of the nitrogen treatment groups (LN_1_ and RN_1_) were significantly higher than those of the non-nitrogen treatment groups (LN_0_ and RN_0_) (*P* < 0.05) (Figure 1). P-Atom^13^C and P-Atom^15^N were significantly and positively correlated with S-Atom^13^C, S-Con^13^C, S-Atom^15^N, and S-Con^15^N (*P* < 0.05) (Supplementary Table 5). The S-Atom^15^N and S-Con^15^N values of the soil in the LN_1_ treatment were the highest and were significantly higher than those in the RN_1_ treatment. These results indicate that at the same temperature level, *M*. *sieversii* plants had a strong ability to exchange with soil under room temperature and nitrogen application, which facilitated the uptake of carbon and nitrogen by seedlings. More photosynthetic products made by the leaves were transported downward to the soil, which provided the material basis for root growth and development, thus alleviating the effects of low temperature stress (Jiang et al., 2015; Cornic, 2022).

### Rhizosphere Soil Enzyme Activity

Soil enzymes are an important indicator of soil biological activity, and all biochemical activities in soil are performed under the action of soil enzymes (Utobo and Tewari, 2015; Nannipieri et al., 2018). Soil enzyme activity is influenced by soil temperature, soil nutrients, microbial community, fertilization, and other factors (Cheng et al., 2013; Díaz et al., 2021; Levakov et al., 2021; Tan et al., 2021). The activities of nitrogen cycle enzymes such as urease, protease, glutaminase, and catalase varied significantly under different nitrogen fertilizer treatments (Cao et al., 2014; He et al., 2021; Li et al., 2021b). Seasonal low temperatures or diurnal variations in temperature can have a significant impact on soil enzymes (Viswanathan and Krishnan, 1962; Cao R. et al., 2021). In a field experiment with a winter temperature range of 0.5–2.0°C, the activities of soil catalase, urease, and phosphatase were reduced by 0.08–1.20 mL g^–1^, 0.004–0.019 mg g^–1^, and 0.10–0.25 mg kg^–1^, respectively (Xiao et al., 2012). In this study, the soil urease, protease, glutaminase, and catalase activities were higher in the R treatment than in the L treatment, and the soil urease, glutaminase, and catalase activities was significantly higher in the N_1_ treatment than in the N_0_ treatment (*P* < 0.05) (Figure 2). These results indicate low temperature significantly reduced the soil urease, protease, and glutaminase activities, while nitrogen application mitigated the effect of low temperature on the activities of nitrogen metabolism related enzymes.

### Rhizosphere Soil Bacterial Community Structure

Soil microorganisms are sensitive to environmental changes, and their composition and activity are influenced by a variety of factors including fertilizations, climate, and plant type (Dong et al., 2014; Soman et al., 2017; Grosso et al., 2018; Hu et al., 2019). Nitrogen fertilizer is an important factor that affects soil microbial communities in many agricultural systems (Wang L. et al., 2021; Zhang X. et al., 2021; Hu et al., 2022). In this study, the bacterial community diversity and richness of the apple rhizosphere soil in the nitrogen application treatment were higher than those without nitrogen treatment. The dominant phyla (relative abundance > 0.5%) of soil bacteria in the different treatments were Proteobacteria, Bacteroidetes, Acidobacteria, and Verrucomicrobia, followed by Firmicutes and Gemmatimonadetes (Figure 3). This result is similar to the dominant bacterial taxa obtained by Joa et al. (2014) and Wu et al. (2020). The higher abundance of the phyla Acidobacteria and Verrucomicrobia in the soil of the room temperature treatment group indicated that the application of nitrogen at room temperature could provide a good survival environment for Acidobacteria and Verrucomicrobia. The major reason is that nitrogen fertilization provides mineral elements for plant growth, promotes the growth and substance secretion of plant root organs, and accordingly increases the physiological activity of the root system (Hamm et al., 2016; Chen et al., 2020). Therefore, nitrogen application increased the relative abundance of Acidobacteria and Verrucomicrobia, which are closely related to the rhizosphere effect.

Microbial photosynthesis plays an important role in agricultural soils, and increased fertilizer application can significantly affect soil carbon decomposition and CO_2_ emissions (Xun et al., 2016; Carrara et al., 2018; Tian et al., 2019). The microorganisms involved in CO_2_ fixation are gram-negative bacteria, with the main dominant group being Proteobacteria (Li et al., 2020; Wang X. et al., 2021). The *Alphaproteobacteria* phylum mainly includes some typical carbon-fixing genera, such as *Rhodopseudomonas* and *Methylibium* (Liao et al., 2020; Chen et al., 2021a). *Bradyrhizobium* is a parthenogenic nitrogen-fixing bacterium that supports nutrient growth by depleting soil resources through fertilizer application (Li et al., 2019). In addition, this genus is found in *Alphaproteobacteria*, and it is usually classified as a eutrophic organism (Jabir et al., 2021). Short-term applications of nitrogen fertilizer can increase the abundance of biological nitrogen-fixing bacteria, and these microbial communities may use the resources in the fertilizer to support their own nutritional growth (Karlidag et al., 2007; Vitousek et al., 2013; Liu et al., 2022). Our results were consistent with the findings of the previous studies mentioned above. In this study, the relative abundances of *Rhodopseudomonas* and *Methylibium* were higher in the R treatment than in the L treatment at the same level of nitrogen application, and the relative abundance of *Bradyrhizobium* was significantly higher in the N_1_ treatment than in the N_0_ treatment at the same temperature level (*P* < 0.05) (Table 2). These results indicate that applying nitrogen fertilizer at the appropriate temperature can increase the number of soil carbon- and nitrogen-fixing bacterial genera. This study did not investigate the role of carbon- and nitrogen-fixing bacteria in the rhizosphere soil material cycle. It is necessary to further quantify the carbon fixation and nitrification characteristics of *Rhodopseudomonas*, *Methylibium*, and *Bradyrhizobium*, which are more responsive to low temperature and nitrogen application than other bacterial genera.

### Relationship Between ^13^C and ^15^N Abundance and Rhizosphere Soil Bacterial Communities

Soil environmental factors have an effect on the soil microbial community (Liu et al., 2021; Wang M. et al., 2021; Yin et al., 2021). Several studies have shown that soil microbial community composition is influenced by NO_3_^–^, soil organic carbon, and soil nitrogen content (Chen et al., 2021c; Liu et al., 2021; Ren et al., 2021). Shen et al. (2015) discovered that the structure of the bacterial community was significantly correlated with soil total carbon, total nitrogen, C:N ratio, and dissolved organic carbon. In this study, P-Atom^15^N and P-Con^15^N showed a significant positive correlation with Verrucomicrobia and Firmicutes (*P* < 0.05) (Figure 5A and Supplementary Table 3), and P-Atom^13^C, P-Con^13^C, P-Atom^15^N, and P-Con^15^N showed a highly significant positive correlation with the carbon and nitrogen metabolism-related genera *Rhodopseudomonas*, *Methylibium*, and *Bradyrhizobium* (*P* < 0.01) (Figure 5B and Supplementary Table 4). Therefore, plant carbon and nitrogen accumulation are key factors affecting the diversity and structure of the rhizosphere soil bacterial community in *M*. *sieversii*. The improvement of nutrient uptake by plants may be related to the mechanisms produced by rhizosphere soil microorganisms (Jacoby et al., 2017; Jing et al., 2021).

## Conclusion

In conclusion, nitrogen application altered rhizosphere soil bacterial communities by influencing soil carbon and nitrogen accumulation as well as enzyme activities related to nitrogen metabolism. Furthermore, nitrogen application aided in the diversification and richness of the bacterial community, as well as the aggregation of carbon- and nitrogen-fixing bacterial genera (*Rhodopseudomonas*, *Methylibium*, and *Bradyrhizobium*) in the rhizosphere soil. RDA suggested that P-Con^13^C and P-Con^15^N were the key variables regulating the composition of the rhizosphere soil bacterial communities in *M*. *sieversii*. This study creates a suitable soil environment for *M*. *sieversii* roots from the perspectives of soil carbon and nitrogen cycling and microbial ecology, which has important practical significance for the breeding of *M*. *sieversii* seedlings and the conservation of *M*. *sieversii* germplasm resources. In the future, more emphasis could be placed on the role and function of carbon- and nitrogen-fixing bacteria in the rhizosphere soil material cycle of *M*. *sieversii*.

## Data Availability Statement

The original contributions presented in the study are included in the article/Supplementary Material, further inquiries can be directed to the corresponding author/s.

## Author Contributions

HZ, SY, and KY planned and designed the study. HZ performed experiments and wrote original draft. FP and FZ commented on data interpretation and the whole manuscript. All authors contributed to the study and approved the final manuscript.

## Conflict of Interest

The authors declare that the research was conducted in the absence of any commercial or financial relationships that could be construed as a potential conflict of interest.

## Publisher’s Note

All claims expressed in this article are solely those of the authors and do not necessarily represent those of their affiliated organizations, or those of the publisher, the editors and the reviewers. Any product that may be evaluated in this article, or claim that may be made by its manufacturer, is not guaranteed or endorsed by the publisher.
