# Supplementary material for: Effects of Temperature and Nitrogen Application on Carbon and Nitrogen Accumulation and Bacterial Community Composition in Apple Rhizosphere Soil
Source: Front Plant Sci. 2022 Apr 4;13:859395. doi: 10.3389/fpls.2022.859395 (PMC9014127; doi:10.3389/fpls.2022.859395)
Supplement: Supplementary file 1 [file Data_Sheet_1.docx]

| Indices | *df* | *F-*value | *P-*value |
| --- | --- | --- | --- |
| **Plant ^13^C and ^15^N abundance** |  |  |  |
| P-Atom^13^C | 3 | 2,344.800 | < 0.001 |
| P-Con^13^C | 3 | 10.552 | 0.004 |
| P-Atom^15^N | 3 | 69.870 | < 0.001 |
| P-Con^15^N | 3 | 693.370 | < 0.001 |
| **Soil ^13^C and ^15^N abundance** |  |  |  |
| S-Atom^13^C | 3 | 3.354 | 0.076 |
| S-Con^13^C | 3 | 3.400 | 0.074 |
| S-Atom^15^N | 3 | 278.170 | < 0.001 |
| S-Con^15^N | 3 | 106.320 | < 0.001 |
| **Soil enzymatic activity** |  |  |  |
| Urease activity | 3 | 1,824.800 | < 0.001 |
| Protease activity | 3 | 339.530 | < 0.001 |
| Glutaminase activity | 3 | 621.400 | < 0.001 |
| Catalase activity | 3 | 17.174 | < 0.001 |
| **alpha diversity index** |  |  |  |
| Shannon | 3 | 88.553 | < 0.001 |
| Simpson | 3 | 34.136 | < 0.001 |
| Chao1 | 3 | 16.999 | < 0.001 |
| Ace | 3 | 26.742 | < 0.001 |
| **bacterial phylum** |  |  |  |
| Proteobacteria | 3 | 8.036 | 0.008 |
| Acidobacteria | 3 | 2.830 | 0.106 |
| Verrucomicrobia | 3 | 4.094 | 0.049 |
| Actinobacteria | 3 | 5.320 | 0.026 |
| **bacterial genus** |  |  |  |
| *Rhodopseudomonas* | 3 | 14.735 | 0.001 |
| *Methylibium* | 3 | 14.087 | 0.001 |
| *Pseudomonas* | 3 | 7.877 | 0.009 |
| *Bradyrhizobium* | 3 | 19.648 | < 0.001 |

**Supplementary Table S1.** The results of a one-way ANOVA for plant ^13^C and ^15^N abundance, soil ^13^C and ^15^N abundance, soil enzymatic activity, alpha diversity index, bacterial phylum and bacterial genus.

Significance levels of one-way ANOVA: *P* < 0.01, highly significant; 0.01< *P* < 0.05, significant; *P* ≥ 0.05, not significant.

**Supplementary Table S2.** Effect of temperature and nitrogen application on significantly changed bacterial phyla in the rhizosphere soil.

| Treatment | Proteobacteria | Acidobacteria | Verrucomicrobia | Actinobacteria |
| --- | --- | --- | --- | --- |
| LN_0_ | 0.726 ± 0.0112a | 0.041 ± 0.0051b | 0.029 ± 0.0064b | 0.019 ± 0.0021ab |
| LN_1_ | 0.672 ± 0.0360b | 0.046 ± 0.0028ab | 0.032 ±0.0009b | 0.027 ± 0.0049a |
| RN_0_ | 0.621 ± 0.0061b | 0.061 ± 0.0140a | 0.040 ± 0.0166ab | 0.017 ± 0.0068b |
| RN_1_ | 0.672 ± 0.0195b | 0.043 ± 0.0011ab | 0.059 ± 0.0066a | 0.010 ± 0.0015b |

Note: Values were shown as means ± standard deviations (SD, n = 3). Different lowercase letters in the same column were indicated statistically significant differences between the four treatments at 0.05 level.

**Supplementary Table S3.** Pearson's correlation coefficients between dominant bacterial phyla and ^13^C and ^15^N abundance.

|  | P-Atom^13^C | P-Con^13^C | P-Atom^15^N | P-Con^15^N | S-Atom^13^C | S-Con^13^C | S-Atom^15^N | S-Con^15^N |
| --- | --- | --- | --- | --- | --- | --- | --- | --- |
| Proteobacteria | -0.018 | -0.265 | -0.073 | -0.004 | -0.133 | 0.098 | -0.057 | -0.063 |
| Bacteroidetes | 0.014 | -0.106 | -0.049 | -0.118 | -0.025 | -0.528 | 0.212 | 0.249 |
| Acidobacteria | -0.312 | 0.087 | -0.292 | -0.321 | 0.024 | -0.125 | -0.224 | -0.228 |
| Verrucomicrobia | 0.421 | 0.587* | 0.597* | 0.621* | 0.743** | 0.398 | 0.056 | 0.029 |
| Firmicutes | 0.588* | 0.524 | 0.591* | 0.586* | 0.365 | 0.525 | 0.419 | 0.370 |
| Actinobacteria | 0.002 | -0.474 | -0.194 | -0.313 | -0.429 | -0.189 | 0.443 | 0.440 |
| Fibrobacteres | 0.034 | 0.012 | 0.255 | 0.193 | 0.446 | -0.140 | -0.067 | -0.072 |
| Gemmatimonadetes | -0.210 | 0.222 | -0.171 | -0.144 | -0.266 | 0.295 | -0.259 | -0.249 |
| Chloroflexi | -0.279 | 0.250 | -0.178 | -0.171 | -0.236 | 0.262 | -0.355 | -0.357 |

Significance levels of one-way ANOVA: *, 0.01 < *P* < 0.05, significant; **, *P* < 0.01, highly significant; ns, *P* ≥ 0.05, not significant.

**Supplementary Table S4.** Pearson's correlation coefficients between significantly changed bacterial genera and ^13^C and ^15^N abundance.

|  | P-Atom^13^C | P-Con^13^C | P-Atom^15^N | P-Con^15^N | S-Atom^13^C | S-Con^13^C | S-Atom^15^N | S-Con^15^N |
| --- | --- | --- | --- | --- | --- | --- | --- | --- |
| *Asticcacaulis* | -0.176 | -0.131 | -0.082 | 0.007 | -0.123 | -0.113 | -0.383 | -0.373 |
| *Devosia* | 0.147 | 0.320 | 0.394 | 0.452 | 0.475 | 0.184 | -0.244 | -0.256 |
| *Rhizobacter* | -0.003 | -0.567 | -0.189 | -0.264 | -0.057 | -0.188 | 0.349 | 0.337 |
| *Ferruginibacter* | 0.245 | -0.180 | 0.107 | 0.037 | 0.144 | -0.322 | 0.519 | 0.541 |
| *Emticicia* | 0.335 | 0.547 | 0.345 | 0.470 | 0.448 | 0.254 | 0.097 | 0.151 |
| *Lacunisphaera* | 0.533 | 0.575 | 0.707* | 0.716** | 0.800** | 0.392 | 0.189 | 0.180 |
| *Pseudolabrys* | 0.246 | 0.364 | 0.394 | 0.463 | 0.574 | 0.063 | -0.059 | -0.038 |
| *Mesorhizobium* | -0.103 | -0.595* | -0.213 | -0.255 | -0.366 | -0.246 | 0.153 | 0.156 |
| *Phenylobacterium* | 0.325 | 0.290 | 0.376 | 0.411 | 0.351 | 0.261 | 0.190 | 0.190 |
| *Dokdonella* | 0.446 | 0.266 | 0.492 | 0.536 | 0.164 | 0.647* | 0.251 | 0.241 |
| *Candidatus_Solibacter* | -0.304 | -0.107 | -0.323 | -0.330 | 0.126 | -0.403 | -0.199 | -0.212 |
| *unidentified_Acidobacteria* | -0.112 | 0.065 | -0.030 | -0.101 | 0.352 | -0.240 | -0.035 | -0.036 |
| *Caulobacter* | 0.635* | 0.081 | 0.583* | 0.543 | 0.364 | 0.299 | 0.593* | 0.604* |
| *Rhodanobacter* | -0.151 | -0.294 | -0.264 | -0.358 | -0.376 | 0.174 | 0.140 | 0.108 |
| *unidentified_Gammaproteobacteria* | -0.442 | 0.017 | -0.388 | -0.325 | -0.488 | -0.079 | -0.560 | -0.594 |
| *Rhodopseudomonas* | 0.813** | 0.837** | 0.851** | 0.901** | 0.682* | 0.683* | 0.463 | 0.440 |
| *Methylibium* | 0.789** | 0.794** | 0.846** | 0.886** | 0.684* | 0.632* | 0.435 | 0.412 |
| *Pseudomonas* | 0.685* | 0.699* | 0.828** | 0.852** | 0.715** | 0.594* | 0.358 | 0.385 |
| *Bradyrhizobium* | 0.863** | 0.782** | 0.896** | 0.911** | 0.749** | 0.674* | 0.557 | 0.526 |

Significance levels of one-way ANOVA: *, 0.01 < *P* < 0.05, significant; **, *P* < 0.01, highly significant; ns, *P* ≥ 0.05, not significant.

**Supplementary Table S5.** Pearson's correlation coefficients between ^13^C and ^15^N abundance and ^13^C and ^15^N abundance.

|  | P-Atom^13^C | P-Con^13^C | P-Atom^15^N | P-Con^15^N | S-Atom^13^C | S-Con^13^C | S-Atom^15^N | S-Con^15^N |
| --- | --- | --- | --- | --- | --- | --- | --- | --- |
| P-Atom^13^C | 1 | 0.593* | 0.927** | 0.906** | 0.685* | 0.622* | 0.853** | 0.844** |
| P-Con^13^C | 0.593* | 1 | 0.632* | 0.727** | 0.605* | 0.676* | 0.202 | 0.199 |
| P-Atom^15^N | 0.927** | 0.632* | 1 | 0.978** | 0.711** | 0.713** | 0.655* | 0.643* |
| P-Con^15^N | 0.906** | 0.727** | 0.978** | 1 | 0.736** | 0.736** | 0.567 | 0.561 |
| S-Atom^13^C | 0.685* | 0.605* | 0.711** | 0.736** | 1 | 0.488 | 0.461 | 0.468 |
| S-Con^13^C | 0.622* | 0.676* | 0.713** | 0.736** | 0.488 | 1 | 0.306 | 0.274 |
| S-Atom^15^N | 0.853** | 0.202 | 0.655* | 0.567 | 0.461 | 0.306 | 1 | 0.995** |
| S-Con^15^N | 0.844** | 0.199 | 0.643* | 0.561 | 0.468 | 0.274 | 0.995** | 1 |

Significance levels of one-way ANOVA: *, 0.01 < *P* < 0.05, significant; **, *P* < 0.01, highly significant; ns, *P* ≥ 0.05, not significant.
